# From Biomechanics to Welfare: Integrative Advances in Equine Sports Medicine and Rehabilitation

**DOI:** 10.3390/ani15182706

**Published:** 2025-09-15

**Authors:** Roberta Blake, David Marlin

**Affiliations:** 1 Agriculture, Animal and Environmental Sciences, Anglia Ruskin University, Lordship Rd, Writtle, Chelmsford CM1 3RR, UK; 2 Animalweb Ltd., The Granary, Hermitage Court, Hermitage Lane, Maidstone ME16 9NT, UK; dm@davidmarlin.co.uk

Equine sports medicine continues to advance at pace, driven by innovations in technology, AI, biomechanics, exercise physiology, and clinical diagnostics. This Special Issue brings together eleven original and review articles that highlight both scientific progress and practical implications for the training, health, and welfare of equine athletes. The breadth of the contributions—ranging from field-applicable motion capture to the influence of tack design—underscores the interdisciplinary nature of modern equine research. Below, we summarize the published work and reflect on the overarching themes that emerge.

## 1. Biomechanics and Modelling Approaches

Kinematics remain central to understanding equine movement. Shaffer et al. presented a markerless motion-capture system capable of providing full-body biomechanical analysis through video and neural-network modelling. By eliminating the need for markers, this technology enables accurate kinematic assessment under real training conditions, enhancing its practical value.

Complementing this, Liedtke et al. proposed a composite model of energy expenditure in cross-country eventing. By integrating heart rate, lactate-based measures of anaerobic contribution, and GPS-derived workload data, their model accounts for terrain and course demands with greater fidelity than single-parameter approaches. These advances illustrate the shift toward multi-modal, field-based monitoring tools that support both performance optimization and welfare oversight.

Biomechanics remain key determinant of athletic performance and soundness. Pizzi et al. evaluated Criollo horses performing traditional manoeuvres such as the esbarrada and volta sobre patas. They showed that static conformation does not predict dynamic performance, reinforcing the value of functional movement analysis.

Finally, a study on effect of girth tension and design on saddle pressures and limb kinematics was published by Marlin et al. Tack design and use are increasingly recognized as welfare and performance relevant variables. Using six regularly ridden horses, they compared straight and anatomical girths at tensions of 8 and 16 kg. While limb kinematics were largely unaffected, increasing girth tension shifted saddle pressure cranially, a finding that may have implications for back discomfort and long-term performance. This work adds valuable empirical evidence to ongoing debates about tack and welfare raised by international equestrian bodies.

## 2. Physiological and Metabolic Insights

Two studies focus on exercise-related physiology. Zhang et al. investigated Mongolian horses over a 20 km endurance ride, documenting changes in blood parameters, antioxidant enzyme activity, and metabolomic markers. Their findings suggest that uric acid and L-tyrosine may serve as indicators of oxidative imbalance, offering potential for early detection of overtraining.

In a different clinical context, Lensing et al. explored the relationship between dental health and gastric pH. While no clear association was found, horses with moderate to severe dental pathology tended to have lower gastric pH, highlighting the need to consider oral health as part of a comprehensive welfare assessment.

## 3. Conformation, Symmetry, and Performance

From a breeding perspective, the feature paper by Ripollés-Lobo et al. assessed conformational defects in Menorca Purebred horses. They found high prevalence and significant heritability for several traits, providing breeders with essential data for selection strategies aimed at long-term soundness.

At the clinical level, Pfau et al. examined the effect of diagnostic anesthesia on gait symmetry. Their work highlights how changes in head and pelvic movement following nerve blocks can inform, but also complicate, the interpretation of lameness assessments.

The review by Haussler et al. addressed the conceptual distinction between laterality and asymmetry. They caution against equating all asymmetrical movement with pathology, reminding clinicians and trainers that lateralization can represent a normal neurobiological phenomenon. This perspective is especially pertinent as technologies now allow increasingly precise measurement of gait asymmetry; interpretation must remain nuanced.

## 4. Respiratory Health and Diagnostics

Respiratory function is a recurring theme in this Special Issue, reflected in three complementary papers. The review by Lendl et al. examined current diagnostic approaches to equine asthma, noting how exercise state, environment, and disease remission complicate interpretation. They emphasize the need for multimodal, context-sensitive diagnostic frameworks.

Adding new clinical data, Lendl et al. have then investigated the Influence of a Standardized Lunging Exercise Test on BALF Cytology in Horses with Mild–Moderate Asthma. Their results showed that exercise prior to bronchoalveolar lavage increased neutrophil proportions in mildly affected horses, significantly improving diagnostic yield. This practical approach may assist in earlier identification of subtle cases, enabling timely intervention.

Together, these works highlight both the conceptual refinement and practical innovation required to improve asthma diagnosis in the field.

## 5. Integrative Themes and Future Directions

Several themes unite these diverse contributions. First, there is a clear trend toward non-invasive, field-applicable diagnostics—from markerless biomechanics to exercise-enhanced asthma testing. These methods promise to bring cutting-edge science into the practical contexts where horses are trained and treated.

Second, the value of integrated models is evident. Studies that combine physiological data, genetic analyses, and kinematic assessments provide a richer understanding than isolated measures. This integrative perspective reflects the complexity of equine performance, where health, environment, and equipment interact.

Third, the welfare dimension runs consistently through this Special Issue. Whether considering oxidative stress in endurance horses, pressure shifts from girth tension, or diagnostic clarity in asthma, each study emphasizes the balance between maximizing performance and safeguarding the horse’s wellbeing.

Finally, both reviews remind us that conceptual clarity matters: distinguishing lateralization from pathological asymmetry, or recognizing the limits of asthma diagnostics, helps prevent misinterpretation and unnecessary interventions.

## 6. Invitation: Submit to the 2nd Edition of This Special Issue

Building on the success of this collection, we are delighted to invite submissions to the 2nd Edition of Advances in Equine Sports Medicine, Therapy and Rehabilitation in Animals until 31 July 2026. Guest Editors Dr. Roberta Blake and Dr. David Marlin welcome manuscripts exploring emerging, technology-driven approaches to equine care—particularly studies on:

- Artificial intelligence (AI) and machine learning in lameness detection, gait analysis, and injury risk modelling;
- Wearable technologies and biomechanical sensors for the real-time monitoring of locomotion, cardiovascular performance, welfare, and recovery;
- Advanced imaging and diagnostics, including portable and dynamic modalities;
- Data-driven rehabilitation protocols, supported by digital tools and technology to monitor recovery;
- Innovations in regenerative therapy including stem cell applications and tissue engineering.

We encourage submissions from researchers, clinicians, and industry partners offering novel methodologies with high impact.

## 7. Conclusions

This Special Issue illustrates the breadth and vitality of contemporary equine sports medicine. Collectively, the eleven contributions offer methodological advances, clinical insights, and conceptual clarity that will inform research and practice alike. They also highlight the importance of cross-disciplinary collaboration—veterinarians, biomechanists, geneticists, physiologists, and trainers all have roles to play.

As equine sports continue to evolve, so too must our approaches to therapy and rehabilitation. Future work should build on the trends evident here: developing real-time field diagnostics, validating biomarkers of training stress, refining tack to reduce adverse loading, and embedding welfare as a guiding principle. By combining technological innovation with a holistic appreciation of the horse, we can ensure that progress in equine sports medicine serves not only performance, but also the long-term health and dignity of the equine athlete.

As we invite the scientific community to contribute to Volume 2, we reaffirm our commitment to advancing equine health, performance, and welfare through collaborative, technology-forward scholarship.

